# From Grass to Graph: NIRS Calibration for Fatty Acid Profiling in Grass-Raised Beef

**DOI:** 10.3390/foods14162767

**Published:** 2025-08-08

**Authors:** Iris Lobos-Ortega, Mariela Silva, Romina Rodríguez-Pereira, Rodolfo Saldaña, Ignacio Subiabre, Marion Rodríguez, Rodrigo Morales

**Affiliations:** 1 Instituto de Investigaciones Agropecuarias INIA Remehue, Ruta 5 Km 8, Osorno 5290000, Chile; iris.lobos@inia.cl (I.L.-O.); marion.rodriguez@inia.cl (M.R.); 2 Escuela de Medicina Veterinaria, Facultad de Recursos Naturales y Medicina Veterinaria, Universidad Santo Tomás, Talca 3460000, Chile; rominarodriguezmv@gmail.com; 3 Instituto de Investigaciones Agropecuarias INIA Ururi, Magallanes 1865, Arica 1000000, Chile

**Keywords:** NIRS, fatty acid, grass-fed, beef, SFA, MUFA

## Abstract

The fatty acid (FA) profile of beef is a key indicator of nutritional quality. This study assessed the ability of Near Infrared Spectroscopy (NIRS) to predict the FA profile in beef samples from southern Chile. A total of 81 FAs were analyzed, and 38% of the calibration models achieved RPD ≥ 2.5 (Ratio of Performance to Deviation). Strong predictive performance was observed for major FAs, particularly SFA and MUFA, with R^2^p > 0.90 (Coefficient of Determination) for palmitic (16:0). Although PUFA and some CLA isomers showed lower predictive accuracy—likely due to low concentrations and spectral overlap—minor FA such as 9*c*,11*t*-18:2 (CLA, rumenic acid) was accurately predicted. External validation confirmed that 77% of FAs showed no significant differences from gas chromatography, highlighting the robustness of NIRS for most compounds analyzed here. NIRS effectively captured FAs related to grass-based diets, such as trans-vaccenic acid and specific CLA isomers. NIRS works as a practical, rapid, and non-destructive tool for FA profiling, with potential uses in nutritional labeling and quality control; however, its application depends on the prior development of robust calibration models, which must be tailored to the specific matrix and analytical objectives.

## 1. Introduction 

The nutritional profile of fatty acids (FAs) in food has a significant effect on human health. Fatty acids, including saturated (SFAs), monounsaturated (MUFAs), and polyunsaturated fatty acids (PUFAs), are essential in several metabolic processes, serving as structural components of cell membranes and precursors to hormones [1]. Meat, particularly beef, is a significant source of those fatty acids, which contribute to overall diet quality. Ruminant meat is especially rich in beneficial omega-3 compounds, including docosapentaenoic acid (DPA), docosahexaenoic acid (DHA), and eicosapentaenoic acid (EPA), which are associated with reduced risks of chronic diseases such as cardiovascular conditions and cognitive decline [2,3]. In addition, ruminant meat contains bioactive compounds such as conjugated linoleic acid (CLA, 9*c*,11*t*-18:2) and trans-vaccenic acid (TVA, 11*t*-18:1), which have been shown to offer health benefits including anti-carcinogenic and anti-inflammatory properties [4,5,6]. Those positive effects are more pronounced in grass-fed cattle, which typically produce meat that has a higher n-3 PUFA content and a more favorable n-6:n-3 ratio than do grain-fed cattle [3,4,7].

Feeding practices play a crucial role in shaping the fatty acid composition of beef [4]. In Chile, cattle farming is predominantly concentrated in the central-southern regions, where favorable climatic conditions and extensive grasslands support grass-based production systems [8]. Those systems, which are based on natural grazing, are environmentally sustainable and produce meat that has a more healthful fatty acid profile, including higher omega-3 content, a more favorable n-6:n-3 ratio, and higher CLA 9*c*,11*t*-18:2 content than do other systems [9,10,11]. Although these systems often result in lower intramuscular fat (IMF) content, the type of forage available can substantially influence both IMF levels and the specific fatty acid composition of the meat [12]. In Chile, gaining a better understanding of these relationships is essential for evaluating the nutritional quality of beef and promoting more sustainable cattle production practices. The fatty acid profile of meat is a key indicator of both its nutritional value and the sustainability of the production system.

Given the nutritional importance of fatty acids, a precise measure of the full fatty acid profile of ruminant meat is crucial [13,14]; however, that is particularly challenging because of the complexity of these compounds, which have required gas chromatography (GC) for their analysis, traditionally [15,16]. Although GC is highly accurate, it is time-consuming, expensive, and environmentally unfriendly, and involves labor-intensive processes, such as lipid extraction and *trans* methylation to produce fatty acid methyl esters (FAMEs). Furthermore, typically, high reproducibility in GC requires multiple runs per sample, and the use of long capillary columns and complex temperature programs further extends analysis times [16]. Correct separation of fatty acid isomers requires specialized columns and carefully optimized chromatographic conditions [17]. Reagent selection and method optimization have a significant influence on the reliability of results [15].

To address those limitations, Near-Infrared Spectroscopy (NIRS) has emerged as a rapid, cost-effective, and non-destructive alternative to GC. NIRS requires minimal sample preparation and can quantify multiple parameters simultaneously [18,19]. Furthermore, NIRS has been extensively used for predicting the quality variables in food, including the fatty acid profile of meat [2,20]. Models developed using lyophilized (freeze-dried) meat have shown better calibration precision than those using fresh meat, because removing water improves the interpretation of the spectral data [2,21]. Nevertheless, challenges remain in fully capturing the complexity of the fatty acid profile in ruminant tissues. Although studies have investigated various sample preparation methods to improve NIRS predictions, the diversity of fatty acids—particularly those present in minor concentrations—and the influence of water content present challenges for achieving precise profiling [22]. There is still limited knowledge about beef samples from pasture-based production systems, which usually have low intramuscular fat and a complex fatty acid profile. To date, no NIRS calibration models have been specifically developed or validated for such conditions, revealing a significant gap in this field. Therefore, the objective of this study was to evaluate the feasibility of NIRS for predicting the complete fatty acid profiles of lyophilized beef samples from southern Chile, which has practical applications for the meat industry.

## 2. Materials and Methods

### 2.1. Collection of Meat Samples

A total of 543 samples from grass-raised beef—specifically the *Longissimus thoracis et lumborum* (LTL) muscle—were purchased from butcher shops in four regions of southern Chile in October 2018 and January 2019. Only butcher shops that sourced animals locally were selected, i.e., butcher shops that bought animals from other regions and sold imported meat were discarded from the study. Immediately after purchase, all samples were placed in portable coolers and transported at 4 °C to the laboratory to preserve their integrity. The measures were conducted during the spring and summer period when cattle, from regions involved in the study, are predominantly pasture-finished [10,23]. The regions included were Araucanía (specifically, the cities of Temuco, Gorbea, Villarrica, and Loncoche), Los Ríos (Lanco, Máfil, Valdivia, Paillaco, Río Bueno, and La Unión), Los Lagos (Osorno, Llanquihue, Puerto Varas, Puerto Montt, Ancud, and Castro), and Aysén (Coyhaique and Aysén). The sampling area (38°45′ S, 72°36′ W to 45°24′ S, 72°42′ W) covered the main grass-fed beef production zones in southern Chile, and together those regions account for 73% of Chile’s total beef production [23,24]. Once in the laboratory, the steaks were frozen at −20 °C and then lyophilized in an ESCO FDL-5L8 freeze-dryer (Esco Technologies Inc., Hatboro, PA, USA). The freeze-dried samples were stored in a desiccator until fatty-acid analysis and subsequently ground in an MM200 mixer mill (Retsch GmbH, Haan, Germany).

### 2.2. Analysis of Total FA Composition

Lipids were extracted from 1 g of freeze-dried and homogenized LTL muscle in a chloroform–methanol mixture (1:1, *v*/*v*). The ground samples were weighed into glass tubes, and the extraction solvent was added to solubilize both neutral and polar lipids. The mixture was vortexed and left to stand at room temperature to allow full lipid partitioning, and then the organic phase was collected. This protocol ensures efficient lipid recovery while preventing degradation or oxidation during handling. Lipid aliquots (~10 mg) from each steak were methylated separately by acidic (methanolic HCl) and basic (sodium methoxide) catalysis to ensure complete methylation of all lipids and avoid isomerization of conjugated linoleic acid (CLA), respectively [13,14]. For quantitative purposes, 1 mL of internal standard (1 mg/mL of 23:0 methyl ester, n-23-M from Nu-Chek Prep Inc., Elysian, MN, USA) was added before methylation. The contents of FA methyl esters (FAMEs) were expressed as a proportion (%) of the total quantified FAMEs. The FAMEs were analyzed by a gas chromatograph (GC) that was equipped with a flame ionization detector (GC-2010 Plus; Shimadzu^®^, Kyoto, Japan). A 100 m SP-2560 ionic liquid column (Supelco, Bellefonte, PA, USA) was operated at two complementary GC temperature programs that plateaued at 175 °C and 150 °C [14]. In addition, another 100 m SLB-IL111 ionic liquid column (Supelco, Bellefonte, PA, USA) was used to confirm the identification of several biohydrogenation intermediates, such as CLA isomers and others [25]. For both columns, hydrogen was the carrier gas at a constant flow rate of 1 mL/min, with the injector and detector temperatures set at 250 °C.

For peak identification, two reference standards (GC #463 and #603), individual FAMEs (21:0, 23:0, 26:0), and a CLA mixture (9*c*,11*t*-/8*t*,10*c*-/11*c*,13*t*-/10*t*,12*c*-/8*c*,10*c*-/9*c*,11*c*-/10*c*,12*c*-/11*c*,13*c*-/11*t*,13*t*-/10*t*,12*t*-/9*t*,11*t*-/8*t*,10*t*-18:2; UC-59M) were used (Nu-Chek Prep Inc., Elysian, MN, USA). Isomerized mixtures of linoleic (18:2n-6) and linolenic (18:3n-3) acids were purchased from Sigma-Aldrich, Bellefonte, PA, USA and branched-chain FAs (BCFA) were identified by a bacterial FAME mixture from Matreya (Pleasant Gap, PA, USA). Several of the trans-18:1 and CLA isomers, along with other non-conjugated dienes not included in the standard mixtures, were identified based on their retention times and elution orders [14,25,26,27,28,29,30,31,32], and were confirmed based on FAME fractions obtained from Ag^+^−SPE cartridges [14,33,34].

All results, including those presented in tables and figures, are expressed on a fresh weight basis.

### 2.3. NIRS and Chemometric Analysis

**Figure 1:**
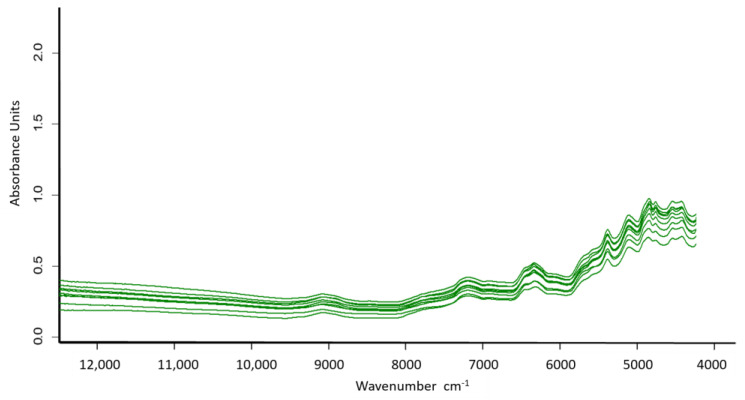
Absorption spectra of freeze-dried samples of beef meat (n = 10) from southern Chile in the NIR range of 12,000 to 4000 cm^−1^.

For NIRS analysis, 10 g of freeze-dried ground beef was placed into a cylindrical glass vial (35 mm internal diameter, 10 mm depth) and scanned in reflectance mode using a BRUKER FT-NIR MPA spectrometer (Bruker Optik GmbH, Ettlingen, Germany). The spectral data were transformed into absorbance (A) by the equation A = log_10_(1/R), where R is the reflectance at each wavenumber between 12,000 and 4000 cm^−1^ (≈750–2500 nm) in the NIR region, using a spectral resolution of 16 cm^−1^ and averaging 64 scans per sample (Figure 1). The use of 64 scans is based on manufacturer recommendations (Bruker Optik GmbH, Ettlingen, Germany) for optimal signal-to-noise ratio in agrifood matrices. A total of n = 543 samples were used, of which 272 were assigned to the calibration set and 271 to the external validation set. Partial least squares regression (PLSR) with test-set validation was used for calibration. The PLSR algorithm selected successive orthogonal factors that maximized the covariance between the predictor variables (spectra) and the response variables (laboratory data). PLSR provides more stable and reliable regression equations and predictions, as it compresses the variables by removing unrelated or unstable data—such as noise and redundancy—while retaining most of the meaningful information [35]. The software OPUS™ version 8.2 (Bruker Optik GmbH, Ettlingen, Germany) was used to create models by selecting wavelengths, mathematical pretreatments, PLSR factors, outlier detection, and regression parameters, among other modeling components. To develop and validate the regression models for each parameter, the samples were divided into two sets: a calibration set for model development and a validation set for testing. To define these two groups, samples were sorted according to the experimental value of each parameter and then alternately assigned—one to calibration and the next to validation. Both sets covered the entire range of chemical values [36].

To optimize calibration accuracy, spectral data were subjected to various preprocessing transformations, including common scatter correction methods and derivations of the original spectrum, in order to enhance adherence to Beer’s law, which states that absorbance and concentration are linearly correlated [37]. The tested preprocessing techniques included vector normalization (VN), minimum-maximum normalization, multiplicative scatter correction (MSC), straight-line subtraction, constant offset elimination, first and second derivatives, or combinations thereof. Outliers were identified and removed during the calibration process, as they could affect model performance and reduce precision for the remaining samples. A maximum of two outlier elimination passes (T and H) were performed before completing the final calibration. T outliers corresponded to samples showing significant discrepancies between their laboratory and predicted values, whereas H outliers were samples whose spectra were too distant (H > 3) from the spectral center of the calibration set [36].

Another important factor in a chemometric model is the number of PLS factors. The selection of these factors is critical to the quality of the analysis. For example:

(i) Choosing too few factors may result in an insufficient explanation of the variation in the spectral and concentration data (underfitting);

(ii) Conversely, choosing too many factors can cause the model to account for minor variations such as spectral noise (overfitting).

Therefore, each PLS model has an optimal number of factors that minimizes the prediction error. In this study, the maximum number of PLS factors was limited to 10 [36].

The root mean square error of calibration (RMSEC) was used to calculate the analysis error of the calibration values (Equation (1)).

(1) $$RMSEC=\sqrt{\frac{\sum_{i=1}^{n}{\left(y_{i}-{\hat{y}}_{i}\right)}^{2}}{n-r-1}}$$

where ŷ_i_ is the NIR predictive value, y_i_ is the chemical reference value of sample i, n is the number of samples and r is the number of PLS factors.

The validation set was used to identify the best preprocessing technique and select the optimal number of PLS factors for each model. The root mean square error of prediction (RMSEP), which represents an objective assessment of the overall error between modeled and reference values, was used to evaluate and compare the accuracy of the different PLS models developed (Equation (2)).

(2) $$RMSEP=\sqrt{\frac{\sum_{i=1}^{n}{\left(y_{i}-{\hat{y}}_{i}\right)}^{2}}{n}}$$

where ŷ_i_ is the NIR predictive value, y_i_ is the chemical reference value of sample i from the prediction set of samples, and n is the number of samples.

Several indicators help determine the optimal number of factors for a given model. The mean prediction error reaches a minimum at the optimal number of PLS factors, while the coefficient of determination (R^2^) reaches a maximum. Thus, the optimal number of PLS factors for a given calibration model can be identified as follows: first, the R^2^ values and mean prediction errors are calculated; then, these values are plotted as a function of the number of PLS factors. The number of factors is considered optimal when these metrics reach their respective extrema and/or show no significant improvement with additional factors. If multiple factor numbers yield similarly good results, it is advisable to select the model with the fewest PLS factors.

Finally, the best models were selected based on the lowest RMSEP. Among the five top-performing models, the one with the lowest number of PLS factors was chosen. The coefficient of determination for prediction (R^2^p) and the residual predictive deviation (RPDp) were also used to evaluate the predictive ability of the models. RPD is defined as the ratio between the standard deviation (SD) of the reference values and the standard error of prediction; it serves as a qualitative indicator of model performance. The smaller the prediction error relative to the variance of the reference values, the higher the RPD value—and consequently, the better the model. An RPD value below 2.4 indicates a poor model, whereas values around 2.5–3.0 may be acceptable for screening purposes [38,39]. In addition to developing NIRS calibration curves, an external validation was conducted based on a random subset of 18 beef samples from various regions in southern Chile, which were not included in the calibration model validation process. For that purpose, the samples were analyzed by GC and NIRS. The predictions obtained from NIRS were compared statistically to the GC reference values by a paired Student’s *t*-test performed in Infostat 2020I software.

## 3. Results

### 3.1. Meat Fatty Acid Composition

Eighty-one fatty acids were detected, and four FA groups were calculated. Descriptive statistics for individual and grouped fatty acids (FA) are presented in Table 1. In the samples of grass-raised beef from southern Chile, the total fatty acid methyl esters (Σ FAME) quantified in intramuscular fat were 2695 mg and 2826 mg, for the calibration and validation sets, respectively. Saturated fatty acids (Σ SFA; 45%), monounsaturated fatty acids (Σ MUFA; 44%), polyunsaturated fatty acids (Σ PUFA; 6%), and conjugated linoleic acids (Σ CLA; 0.7%) were detected (Table 2). Other minor groups, such as non-conjugated dienes (0.7%), trienes (0.2%), and dimethyl acetals (Σ DMA; 2.9%), were also identified.

Myristic (14:0), palmitic (16:0), and stearic acid (18:0) were the most abundant FAs and, along with MUFA 9*c*-16:1, 9*c*-18:1/10*c*-18:1, and the PUFA 18:2n-6, accounted for 82% of Σ FAME. The co-elution of oleic acid (9*c*-18:1) and 10c-18:1 was 35% of Σ FAME. Among MUFA, *trans*-vaccenic acid (11*t*-18:1), a key intermediate in biohydrogenation, was the third-most abundant FA (1.7%).

Within PUFA, linoleic acid (18:2n-6) had the highest proportion (2.6%), followed by alpha-linolenic acid (18:3n-3) at 1.1%. Among omega-3 FAs, eicosapentaenoic acid (EPA, 20:5n-3), docosapentaenoic acid (DPA, 22:5n-3), and docosahexaenoic acid (DHA, 22:6n-3) collectively accounted for nearly 20% of PUFA in the meat samples. Although rumenic acid (RA; 9*c*,11*t*-18:2) represented only 0.5% of total FA, it accounted for 75% of the CLA isomers detected in beef fat. This predominance within the CLA group highlights its important biological role, despite its low concentration in the overall human diet.

### 3.2. NIRS Models

#### 3.2.1. NIR Spectral Features

The mean spectrum began at approximately 0.4 absorbance units (with a minimum value above 0.2), which was sustained up to 9400 cm^−1^, and ended at 4800 cm^−1^ with values around 0.7 and a maximum close to 1.0. The average NIR absorbance spectrum of the freeze-dried meat samples exhibited two main bands, with maxima at 7500–6400 cm^−1^ and 5400–4900 cm^−1^, corresponding to the O–H first overtone and the O–H combination band, respectively (Figure 1). Other spectral features associated with C–H bonds in lipids were also detected, including a second overtone (~8248 cm^−1^), a first overtone (~5779 cm^−1^ and ~5668 cm^−1^), and combination bands (~4300 cm^−1^).

#### 3.2.2. NIRS Calibration and Validation

**Figure 2:**
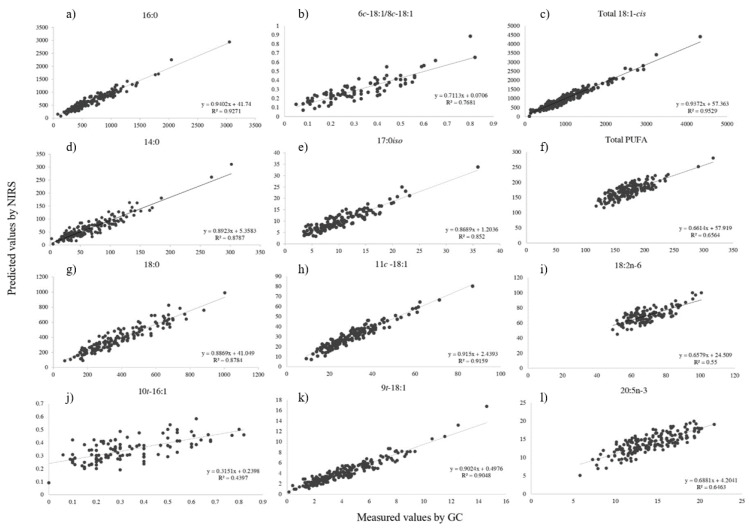
Linear regression between NIRS-predicted and GC-measured values (mg 100 g^−1^ meat) for: (**a**) 16:0; (**b**) 6*c*-18:1/8*c*-18:1; (**c**) Σ 18:1-*cis*; (**d**) 14:0; (**e**) 17:0*iso*; (**f**) Σ PUFA; (**g**) 18:0; (**h**) 11*c*-18:1, (**i**) 18:2n-6; (**j**) 10*t*-16:1; (**k**) 9*t*-18:1; (**l**) 20:5n-3; in beef samples.

**Figure 3:**
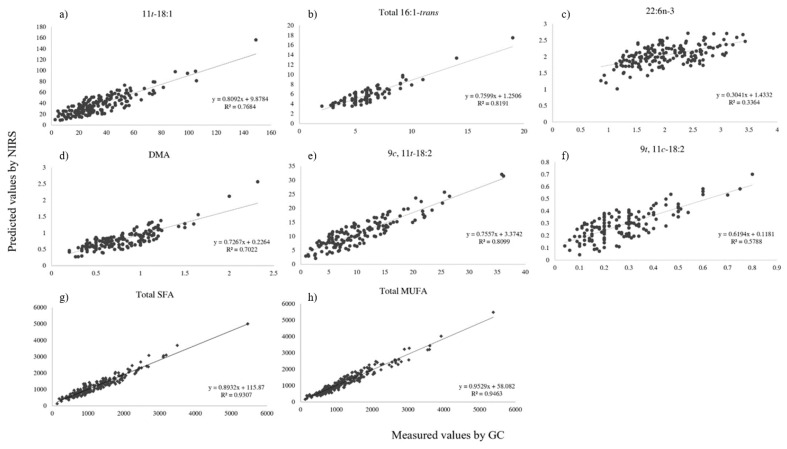
Linear regression between NIRS-predicted and GC-measured values (mg 100 g^−1^ meat) for: (**a**) 11*t*-18:1; (**b**) Σ 16:1-*trans*; (**c**) 22:6n-3; (**d**) DMA; (**e**) 9*c*-11*t*-18:2; (**f**) 9*t*,11*c*-18:2; (**g**) Σ SFA; (**h**) Σ MUFA; in beef samples.

The predictive capacity of NIRS for fatty acid profiling in beef samples is presented in Table 2. After testing several mathematical treatments, the best calibration model was selected based on the R^2^, RMSEP, and RPDp statistics from the validation set. The relationship between NIRS predictions and the reference method results is shown in Figure 2 and Figure 3.

A total of 81 calibration models were analyzed, and 38% of models achieved RPD values ≥ 2.5. The models were able to predict 58% of the MUFAs, 29% of the SFAs, and 3% of the CLAs. The spectral pre-treatments and factors used to develop calibrations and total fatty acids used in this study are shown in Table 2. The number of PLS factors ranged from 1 to 9. The pre-processing treatments used to build the calibration models included: first-order derivative (1D; 36%), no mathematical treatment (NSDP; 36%), standard normal variate (SNV; 15%), SNV combined with first-order derivative (1D+SNV; 12%), and constant offset elimination (COE), which was applied in a single model (18:3n-6).

The coefficients of determination for the validation within Σ FAME ranged from 0.18 (PUFA 20:3n-6) to 0.96 (cis-MUFA 9*c*-18:1/10*c*-18:1). Among FA groups, the highest R^2^p were for Σ MUFA (R^2^p = 0.95) and Σ SFA (R^2^p = 0.93).

The model exhibited excellent calibration performance for most SFA, as reflected by high coefficients of determination in prediction (R^2^p ≥ 0.86) and residual predictive deviation (RPD ≥2.5). Fatty acids with strong predictive capacity included 14:0, 16:0, 17:0, 18:0, and 20:0. Within this group, the BCFA 17:0*iso*, 18:0*iso,* and 17:0*ai*/13*t*-16:1 also demonstrated good calibration metrics (Table 2). Regarding MUFA, similarly robust calibration results were obtained. High R^2^p values (0.95–0.96) and RPD ≥ 2.5 were recorded for Σ 18:1-*cis*, Σ MUFA, and the 9*c*-18:1/10*c*-18:1 isomers. Additional MUFA with strong predictive ability included 16:1-*cis*, 18:1-*cis*, 9*c*-14:1, 7*c*-16:1, 9*c*-17:1, 11*c*-18:1, 13*c*-18:1, 15*c*-18:1, 10*c*-20:1, 11*c*-20:1, 18:1-trans, 9*t*-16:1, 6*t*-18:1/8*t*-18:1, 9*t*-18:1, 12*t*-18:1, and 16*t*-18:1/*c,t*-18:2. Total CLA (Σ CLA) and several non-conjugated dienes such as 9*c*,13*t*-18:2/8*t*,12*c*-18:2, 8*t*,13*c*-18:2, and 9*c*,15*c*-18:2 also fell within this category, indicating their suitability for accurate quantification under the current model.

Moderate predictive capacity was characterized by RPD values between 2.0 and 2.4, indicating acceptable model performance for detection purposes, albeit with reduced precision. Among SFA, this group included 10:0, 12:0, 15:0, and 19:0, as well as BCFA isomers 15:0*iso* and 15:0*ai*. Within the MUFA group, compounds such as 11*c*-16:1, 13*c*-16:1, 6*c*-18:1/8*c*-18:1, 16:1-trans, 11*t*-16:1/12*t*-16:1, 10*t*-18:1, 11*t*-18:1, and 13*t*-18:1/14*t*-18:1, 15*t*-18:1 displayed comparable calibration results. Conjugated linoleic acid (CLA) groups—specifically Σ CLA-*trans,trans* and Σ CLA-*cis,trans*—as well as positional isomers like 7*t*,9*c*-18:2, 9*c*,11*t*-18:2, and 11*t*,13*t*-18:2, also exhibited moderate predictive ability. The triene 9*c*,11*t*,15*t*-18:3 was included in this intermediate group as well.

Fatty acids exhibiting poor predictive performance, characterized by RPD values below 2.0, were considered unsuitable for quantitative prediction, yet useful for coarse classification into concentration levels (i.e., low, medium, or high). Within this group, only one SFA, 22:0, was detected with insufficient predictive accuracy. Several trans-MUFA, including 9*c*-16:1 and 12*c*-18:1, along with positional isomers such as 6*t*-16:1/8*t*-16:1, 10*t*-16:1, and 14*t*-16:1/12*c*-16:1, also showed significantly low RPD values, falling into this category. Additionally, all PUFA displayed limited predictive reliability, preventing their effective quantification. Similar shortcomings were observed for individual isomers of conjugated linoleic acid (CLA), such as 9*t*,11*c*-18:2, 11*c*,13*t*-18:2/21:0, 12*t*,14*t*-18:2, and 7*t*,9*t*-18:2/10*t*,12*t*-18:2. Lastly, various unconjugated dienes and trienes, including 9*t*,12*c*-18:2; 11*t*,15*c*-18:2/10*t*,15*c*-18:2; and 9*c*,11*t*,15*c*-18:3/20:3n-9, were also poorly predicted, confirming the model’s limited applicability to structurally complex or low-abundance PUFA derivatives (Table 2).

#### 3.2.3. External Validation of Fatty Acid Prediction

The comparison between GC and NIRS showed no statistically significant differences (*p* > 0.05) for most FA, indicating a good level of agreement between both analytical methods for those compounds (Table 3). Some FAs, such as 15:0 and 17:0, exhibited moderate predictive capacity (R^2^ = 0.450 and 0.650, respectively), yet differed significantly from GC values (*p* < 0.001), suggesting possible matrix effects or model limitations in absolute quantification. In contrast, long-chain SFAs such as 20:0 and 22:0 showed no significant differences (*p* > 0.05); however, their extremely low R^2^ values (≤ 0.04) indicated limited prediction consistency.

For MUFA, most compounds showed no significant differences between NIRS and GC (*p* > 0.05), suggesting acceptable agreement in absolute values, despite generally low to moderate predictive accuracy (R^2^ ≤ 0.50). One exception was 9*t*-16:1 (R^2^ = 0.55), with moderate predictive performance but still differed significantly from GC values. Conversely, the model performed comparatively better for PUFA, particularly omega-3 species. Fatty acids such as 18:3n-3 (R^2^ = 0.81), Σ n-3 (R^2^ = 0.66), and 20:4n-3 (R^2^ = 0.59) showed good relative predictions, but significant differences with GC values suggest limited accuracy for exact quantification. Minor FA groups such as CLA and non-conjugated dienes/trienes yielded variable results. While Σ CLA was moderately predicted (R^2^ = 0.470, *p* < 0.001), several individual CLA isomers achieved R^2^ values above 0.45 without significant differences from GC, supporting their selective applicability for semi-quantitative or comparative analysis.

## 4. Discussion

The fatty acid (FA) composition of the beef samples analyzed in this study was broadly consistent with reports for grass-finished cattle, both from the same region [12] and from other geographic areas [13,40]. In particular, the high proportions of saturated (Σ SFA) and monounsaturated fatty acids (Σ MUFA), each around 45%, and a polyunsaturated fatty acid (Σ PUFA) content near 6%, aligned with previously described intramuscular fat profiles in beef.

### 4.1. NIRs Prediction

The absorbance values and spectral shapes obtained were similar to those reported for freeze-dried beef by Andueza et al. [20] and Ripoll et al. [41]. Although freeze-dried samples show lower absorbance than fresh meat [2,22], the spectral shape remains comparable across species [42], supporting the applicability of NIRS across different types of meat.

The way fatty acid concentrations are expressed can also influence calibration outcomes. Studies have shown that models based on absolute content (g/kg) often yield better calibration than those using relative percentages, as NIRS operates on Beer–Lambert’s law and responds linearly to concentration [43]. However, good results using percentage data have also been reported in meat matrices [44,45], suggesting that other factors—such as spectral resolution and sample variability—also play a key role.

Pre-processing techniques are typically used to reduce noise, correct baseline shifts, and enhance informative spectral features. In this study, many calibrations performed well without extensive pre-treatment. However, the most effective corrections included first derivatives, which enhanced peak resolution and reduced scattering, and standard normal variate (SNV), which minimized multicollinearity and baseline variation. This is consistent with previous findings in ground freeze-dried meat samples [2,41]. Nonetheless, as emphasized by Lobos-Ortega et al. [46], the quality of reference data and spectral signal remains critical for developing reliable calibration models.

Table 2 summarizes the calibration results for individual fatty acids. High-abundance fatty acids—those typically exceeding 1% of Σ FAME—such as oleic acid, palmitic acid, and stearic acid, achieved strong predictive performance (R^2^p ≥ 0.89), showing that stronger signals and higher concentrations lead to more reliable models. In contrast, low-abundance fatty acids were more affected by prediction error, likely due to lower signal-to-noise ratios and spectral overlap [47]. For example, behenic acid (22:0) and several CLA isomers, present at <1% of Σ FAME, yielded weaker results (R^2^p < 0.70 or RPDp < 2.0), as also noted in previous studies [2,20,41].

Structural complexity, in addition to concentration, plays a critical role in the calibration performance of fatty acids. For instance Σ CLA was well predicted (R^2^p = 0.84), but some individual isomers, such as CLA 9*t*,11*c*-18:2 and 12*t*,14*t*-18:2, showed moderate calibration metrics (R^2^p = 0.57 and 0.69, respectively), reflecting challenges in modeling structurally similar compounds. As suggested by Mourot et al. [22], the spectral similarity of certain double-bond configurations, particularly among conjugated and non-conjugated isomers, can complicate NIRS calibration, especially when sample variability is limited. These overlapping absorption patterns reduce the model’s ability to resolve and distinguish signals. In some cases, even when the model has moderate predictive accuracy, the prediction error remains within an acceptable range. For example, 10:0 had an R^2^p of 0.79 and an RMSEP of 0.4, with an RPDp of 2.2, suggesting that even though the model did not fully capture all sample variability, it still yielded reliable estimates for this low-abundance fatty acid.

Our PUFA models showed improved predictive performance compared to those reported by Giaretta et al. [2] for freeze-dried beef, except for Σ n-3 and Σ n-6 fatty acids. Results were more comparable to those of Mourot et al. [22] and Hernández-Jiménez et al. [48], who improved model performance through expanded datasets with different ruminant species or the use of artificial neural networks, respectively. These studies support the notion that NIRS models generally show moderate to low accuracy (R^2^p ~0.4–0.7) for PUFA in meat and fat, with better outcomes for Σ PUFA than for individual minor components. As such, whereas NIRS has limited quantitative resolution for detailed PUFA profiling, it remains suitable for broader applications such as nutritional labeling, classification of products by fatty acid category, and general screening in food quality control.

The predictive performance for Σ CLA and major intermediates was also robust, whereas moderate accuracy was observed for minor isomers. These findings are in line with Prieto et al. [49,50], who analyzed CLA in subcutaneous and adipose tissues of cattle under different feeding regimes.

Finally, our NIRS models for Σ MUFA and key isomers showed accuracy and robustness comparable to those reported by Pla et al. [45] in rabbit meat from conventional and organic systems. Moreover, our results surpassed those of Giaretta et al. [2], who were unable to establish reliable NIRS models for FA families in both raw and freeze-dried meat.

### 4.2. External Validation

Finally, the robustness of our model was assessed using an independent subset of 18 freeze-dried beef samples not included in the original calibration set. These samples were analyzed by both GC and NIRS, and the predicted values were statistically compared using a paired Student’s *t*-test. The external validation revealed that 77% of the fatty acids evaluated did not show statistically significant differences between NIRS and GC values (*p* > 0.05), indicating satisfactory level of agreemet between both analytical methods. In contrast, the remaining 23% of fatty acids exhibited significant differences (*p* < 0.05), coinciding with those compounds that presented the lowest predictive performance, particularly those with RPDp values below 2.4 (Table 2). These included mainly PUFAs, CLA isomers, and other non-conjugated species, suggesting the need for targeted improvement of the respective calibration models.

Our study showed that partial least-squares regression (PLSR) with test-set validation generally produced consistent results, but divergence was observed for FAs present at low concentrations or with structural characteristics that generate complex spectral patterns. Consequently, some calibrations may require further refinement or expanded sample coverage to improve reliability in new contexts. These challenges were especially evident for certain PUFA and *trans*-isomers, where moderate or low R^2^p values tended to correspond with poor performance in external validation—although exceptions also occurred. In practical terms, this suggests that NIRS can reliably predict major FAs relevant for nutritional labeling and quality control, yet outcomes for minor or complex ones should be interpreted carefully.

To improve model accuracy, especially for minor fatty acids, future efforts should expand the calibration dataset to capture greater variability in breed, diet, muscle type, and processing conditions. In parallel, optimizing key factors such as analyte concentration, tissue composition, sample homogenization, scan number, and scanned surface area will help reduce noise and enhance robustness [42,51]. Controlling these variables will be essential to ensure consistent performance and broader applicability of NIRS-based predictions in meat processing environments. Although freeze-drying improves spectral resolution and model accuracy, its limited compatibility with industrial workflows emphasizes the need to, in future research, focus on developing robust calibrations based on fresh meat samples to support real-time, in-line applications.

## 5. Conclusions

This study demonstrated the feasibility of NIRS for predicting the FA profile of grass-raised beef. The calibration models showed strong predictive performance for major FA groups, particularly SFA and MUFA. In addition, the method proved acceptable for detecting specific biohydrogenation intermediates such as trans-vaccenic acid (11*t*-18:1) and rumenic acid (CLA 9*c*,11*t*-18:2), two fatty acids commonly recognized as important indicators of grass-based production systems. However, whereas these markers provide valuable insights, further refinement of the calibration models is needed to enhance prediction accuracy for additional biohydrogenation products, PUFA, and individual CLA isomers, allowing for a more robust determination of pasture-based origin.

Taken together, the results highlight NIRS as a valuable tool for nutritional labeling and meat quality evaluation, enabling rapid, multi-parameter analysis that enhances transparency and traceability across the beef supply chain. Whereas gas chromatography (GC) remains as the reference method for FA analysis, its complexity and time requirements limit its applicability for routine use. In contrast, NIRS offers a practical and efficient alternative for quality control and compositional screening.

## Figures and Tables

**Table 1:** Descriptive statistics for total fatty acid profiles in grass-raised bovine *Longissimus thoracis et lumborum* muscle samples from southern Chile.

	Calibration (n = 272)				Validation (n = 271)			
Fatty Acid	Mean ± SD	Min	Max	CV	Mean ± SD	Min	Max	CV
	(mg · 100 g^−1^ Meat)			(%)	(mg · 100 g^−1^ Meat)			(%)
**Σ FAME**	2695.1 ± 1493.9	550.3	12,351.9	0.55	2825.9 ± 1516.2	523.7	11,427.3	0.54
**Σ SFA**	1196.3 ± 691.5	154.0	4746.0	0.58	1278.8 ± 755.5	140.0	5462.0	0.59
10:0	1.3 ± 0.9	0.1	7.2	0.68	1.5 ± 1.2	0.1	9.3	0.79
12:0	1.7 ± 1.2	0.2	9.9	0.70	1.8 ± 1.3	0.2	8.0	0.73
14:0	63.9 ± 46.8	2.2	375.3	0.73	69.2 ± 50.8	1.8	301.9	0.73
15:0	10.3 ± 6.6	1.3	46.4	0.65	11.1 ± 7.7	1.1	61.0	0.69
16:0	660.7 ± 397.4	74.3	3014.0	0.60	706.0 ± 423.3	75.6	3051.9	0.60
17:0	23.7 ± 13.9	2.5	79.3	0.59	25.6 ± 16.1	2.5	116.8	0.63
18:0	384.3 ± 213.0	66.7	1343.1	0.55	410.0 ± 241.3	51.4	1636.0	0.59
19:0	1.6 ± 1.0	0.1	6.3	0.65	1.7 ± 1.1	0.1	6.4	0.67
20:0	2.3 ± 1.2	0.5	7.4	0.55	2.6 ± 1.7	0.5	10.9	0.67
22:0	0.9 ± 0.5	0.0	4.7	0.64	0.9 ± 0.6	0.0	4.8	0.68
**Σ BCFA**								
15:0*iso*	4.9 ± 3.4	0.2	20.7	0.69	5.2 ± 4.0	0.3	36.2	0.78
17:0*iso*	10.7 ± 6.6	2.0	50.2	0.61	11.3 ± 6.9	2.0	60.7	0.61
18:0*iso*	2.8 ± 2.1	0.3	17.8	0.74	3.0 ± 2.2	0.3	17.1	0.74
15:0*ai*	5.0 ± 3.4	0.7	26.6	0.68	5.3 ± 3.8	0.5	29.3	0.72
17:0*ai*/13*t*-16:1	15.7 ± 10.4	1.7	77.7	0.66	16.7 ± 10.9	1.6	83.5	0.65
**Σ MUFA**	1198.9 ± 779.0	132.0	6954.0	0.65	1248.9 ± 732.7	128.0	5379.0	0.59
**Σ 16:1-** ***cis***	95.8 ± 77.4	8.0	802.0	0.81	99.1 ± 65.7	8.0	442.0	0.66
**Σ 18:1-** ***cis***	977.8 ± 634.3	109.0	5589.0	0.65	1019.4 ± 593.7	109.0	4333.0	0.58
9*c*-14:1	15.2 ± 16.6	0.4	169.8	1.09	15.8 ± 13.0	0.2	81.1	0.82
7*c*-16:1	5.9 ± 3.9	1.0	39.9	0.66	6.1 ± 3.5	1.2	27.4	0.57
9*c*-16:1	85.6 ± 69.8	6.6	722.0	0.82	88.7 ± 59.9	6.2	394.6	0.68
11*c*-16:1	3.1 ± 3.5	0.0	37.5	1.14	3.1 ± 2.4	0.2	14.0	0.78
13*c*-16:1	1.2 ± 1.3	0.0	13.7	1.11	1.2 ± 1.0	0.0	5.9	0.82
9c-17:1	17.1 ± 11.1	2.3	107.7	0.65	17.8 ± 10.5	2.5	81.7	0.59
6*c*-18:1/8*c*-18:1	0.4 ± 0.4	0.0	3.4	1.06	0.4 ± 0.4	0.0	4.5	1.03
9*c*-18:1/10*c*-18:1	932.9 ± 604.5	98.9	5218.7	0.65	973.3 ± 569.2	101.8	4150.9	0.58
11*c*-18:1	32.1 ± 22.7	8.1	284.0	0.71	32.8 ± 17.5	6.1	126.8	0.53
12*c*-18:1	2.3 ± 1.6	0.3	13.6	0.68	2.5 ± 2.0	0.4	18.3	0.78
13*c*-18:1	6.7 ± 6.5	0.2	67.0	0.98	6.8 ± 4.7	0.3	28.9	0.69
15*c*-18:1	3.3 ± 2.2	0.1	12.6	0.67	3.4 ± 2.6	0.3	17.1	0.76
10*c*-20:1	1.6 ± 1.4	0.1	12.6	0.86	1.7 ± 1.2	0.1	7.8	0.73
11*c*-20:1	2.9 ± 2.8	0.4	35.0	0.97	3.0 ± 2.4	0.2	23.4	0.79
**Σ 16:1-** ***trans***	6.3 ± 3.5	2.0	36.0	0.56	6.4 ± 3.5	1.8	26.0	0.55
**Σ 18:1-** ***trans***	81.6 ± 52.6	9.0	297.0	0.64	84.6 ± 60.5	5.0	398.0	0.72
6*t*-16:1/8*t*-16:1	0.3 ± 0.3	0.0	1.9	0.84	0.4 ± 0.3	0.0	3.1	0.94
9*t*-16:1	3.1 ± 1.7	0.6	10.7	0.55	3.0 ± 1.7	0.3	10.8	0.56
10*t*-16:1	0.4 ± 0.4	0.0	5.1	1.11	0.5 ± 0.6	0.0	7.1	1.27
11*t*-16:1/12*t*-16:1	1.7 ± 1.3	0.0	13.5	0.77	1.7 ± 1.2	0.2	7.8	0.67
14*t*-16:1/12*c*-16:1	0.8 ± 0.9	0.0	11.9	1.12	0.8 ± 0.6	0.1	4.4	0.75
6*t*-18:1/8*t*-18:1	3.2 ± 2.1	0.5	16.0	0.65	3.4 ± 2.5	0.2	17.7	0.74
9*t*-18:1	4.5 ± 2.9	0.4	20.3	0.65	4.7 ± 3.2	0.1	22.6	0.68
10*t*-18:1	4.5 ± 3.7	0.3	26.8	0.84	4.9 ± 5.0	0.2	36.8	1.02
11*t*-18:1	45.7 ± 34.8	5.4	200.9	0.76	46.1 ± 37.9	2.6	216.6	0.82
12*t*-18:1	5.8 ± 3.6	0.8	28.4	0.63	6.2 ± 4.1	0.6	27.5	0.67
13*t*-18:1/14*t*-18:1	10.4 ± 6.7	0.9	40.7	0.64	11.2 ± 8.2	0.9	66.1	0.73
15*t*-18:1	2.1 ± 2.0	0.1	10.6	0.96	2.3 ± 2.6	0.1	22.8	1.12
16*t*-18:1/*c,t*-18:2	5.5 ± 3.7	0.4	20.5	0.68	5.9 ± 4.6	0.2	33.1	0.79
**Σ PUFA**	176.4 ± 37.1	81.0	356.0	0.21	175.3 ± 37.5	80.6	316.0	0.21
**Σ n-6**	108.8 ± 29.6	50.0	245.2	0.27	108.3 ± 28.8	59.0	203.0	0.27
18:2n-6	72.7 ± 22.4	30.8	180.9	0.31	72.3 ± 22.2	33.0	153.9	0.31
18:3n-6	0.7 ± 0.3	0.0	2.6	0.47	0.7 ± 0.3	0.0	2.0	0.48
20:2n-6	0.8 ± 0.4	0.1	2.1	0.46	0.8 ± 0.5	0.2	4.2	0.61
20:3n-6	6.8 ± 1.9	2.6	13.7	0.28	6.7 ± 1.8	2.9	13.2	0.26
20:4n-6	26.1 ± 7.4	8.3	54.4	0.28	25.9 ± 7.1	13.7	53.8	0.27
22:4n-6	1.9 ± 0.9	0.0	6.4	0.47	1.9 ± 0.9	0.0	5.0	0.47
**Σ n-3**	67.5 ± 18.0	12.9	127.7	0.27	66.9 ± 18.2	15.2	128.3	0.27
18:3n-3	30.0 ± 12.0	3.5	83.1	0.40	29.5 ± 11.9	5.3	86.7	0.40
20:3n-3	0.8 ± 0.5	0.0	3.3	0.62	0.8 ± 0.4	0.0	3.5	0.57
20:4n-3	3.6 ± 1.4	0.0	8.6	0.40	3.5 ± 1.4	0.0	8.6	0.39
20:5n-3 (EPA)	14.0 ± 4.4	2.5	29.4	0.32	14.2 ± 4.5	2.2	38.6	0.32
22:5n-3 (DPA)	17.2 ± 3.5	6.2	26.3	0.21	17.1 ± 3.5	6.1	27.1	0.20
22:6n-3 (DHA)	2.0 ± 1.0	0.0	5.3	0.49	1.9 ± 1.0	0.0	5.7	0.50
**Σ DMA**	79.9 ± 14.3	46.0	125.0	0.18	79.4 ± 14.1	39.0	144.0	0.18
**Σ CLA**	18.3 ± 14.5	2.4	102.6	0.79	18.2 ± 13.1	1.7	83.4	0.72
**Σ CLA-** ***trans,trans***	1.9 ± 1.3	0.0	7.3	0.69	2.0 ± 1.5	0.0	10.1	0.76
**Σ CLA-** ***cis,trans***	16.6 ± 13.6	2.2	96.7	0.82	16.4 ± 12.0	1.3	75.2	0.73
7*t*,9*c*-18:2	0.9 ± 0.8	0.1	5.3	0.82	0.9 ± 0.7	0.1	4.9	0.74
9*c*,11*t*-18:2	13.9 ± 11.8	1.6	82.9	0.85	13.6 ± 10.2	0.9	65.1	0.75
9*t*,11*c*-18:2	0.3 ± 0.3	0.0	2.2	0.92	0.3 ± 0.3	0.0	2.0	0.99
11*c*,13*t*-18:2/21:0	0.4 ± 0.3	0.0	2.2	0.78	0.4 ± 0.5	0.0	5.7	1.17
11*t*,13*t*-18:2	1.0 ± 0.7	0.1	4.3	0.73	1.1 ± 0.9	0.1	5.5	0.84
7*t*,9*t*-18:2/10*t*,12*t*-18:2	0.5 ± 0.4	0.0	2.5	0.77	0.5 ± 0.4	0.0	3.5	0.78
12*t*,14*t*-18:2	0.4 ± 0.4	0.0	3.5	1.02	0.4 ± 0.4	0.0	3.3	1.01
**Σ NC-dienes**								
9*c*,13*t*-18:2/8*t*,12*c*-18:2	5.2 ± 3.7	0.5	27.4	0.71	5.4 ± 3.8	0.4	25.9	0.71
8*t*,13*c*-18:2	2.8 ± 2.0	0.2	13.3	0.73	2.9 ± 2.0	0.2	15.6	0.71
9*t*,12*c*-18:2	0.7 ± 0.6	0.0	5.9	0.93	0.6 ± 0.5	0.0	3.8	0.81
11*t*,15*c*-18:2/10*t*,15*c*-18:2	6.8 ± 5.3	0.3	31.6	0.78	6.5 ± 5.3	0.0	35.1	0.82
9*c*,15*c*-18:2	2.4 ± 1.9	0.0	19.2	0.80	2.5 ± 1.7	0.0	12.2	0.67
**Σ NC-trienes**								
9*c*,11*t*,15*c*-18:3/20:3n-9	3.3 ± 1.4	0.4	9.1	0.42	3.3 ± 1.6	0.9	12.4	0.48
9*c*,11*t*,15*t*-18:3	0.8 ± 0.8	0.1	4.8	0.95	0.8 ± 0.6	0.0	3.4	0.84

Σ FAME: Fatty acids methyl ester; Σ SFA: saturated fatty acids; Σ BCFA: branched-chain saturated fatty acid; Σ DMA: dimethyl acetal; Σ MUFA: Monounsaturated fatty acids; Σ PUFA: polyunsaturated fatty acids; ΣCLA: Conjugated linoleic acid; Σ NC: non-conjugated; SD: standard deviation; CV: coefficient of variation.

**Table 2:** NIRS calibration and validation statistics for total fatty acid profiles in grass-raised bovine *Longissimus thoracis et lumborum* muscle samples from southern Chile.

	Mathematical Treatment	#PLS	Calibration			Validation			Frequency Ranges, cm^−1^
			R^2^c	RMSEE	RPDc	R^2^p	RMSEP	RPDp	
**Σ SFA**	NSDP	7	0.92	174.0	3.6	0.93	181.0	3.8	3594.9–12,489.5
10:0	1D+SNV	2	0.80	0.3	2.3	0.79	0.4	2.2	7506–6796.3; 4852.3–4242.9
12:0	1D	2	0.84	0.4	2.5	0.80	0.5	2.3	9403.7–5446.3; 4428–4242.9
14:0	NSDP	5	0.91	13.9	3.3	0.88	15.2	2.9	3594.9–12,489.5
15:0	1D	4	0.84	2.7	2.5	0.83	1.8	2.4	9403.7–8447.2; 5454–4242.9
16:0	1D	3	0.92	109.0	3.5	0.93	104.0	3.7	7506–4242.9
17:0	1D	8	0.88	4.3	3.0	0.86	4.0	2.7	9403.7–6094.3; 4605.4–4242.9
18:0	1D	7	0.90	59.7	3.2	0.88	61.2	2.9	9403.7–544.3; 4605.4–4242.9
19:0	1D	4	0.75	0.4	2.0	0.74	0.4	2.0	5778–5446.3
20:0	SNV	9	0.88	0.4	2.9	0.88	0.4	2.9	6120–4242.9
22:0	1D+SNV	2	0.51	0.2	1.4	0.56	0.2	1.6	6102–4597.7
**Σ BCFA**									
15:0*iso*	1D+SNV	7	0.86	1.2	2.7	0.82	1.0	2.4	9403.7–5446.3; 4428–4242.9
17:0*iso*	NSDP	5	0.89	2.2	3.0	0.85	1.8	2.6	3594.9–12,489.5
18:0*iso*	1D	4	0.87	0.5	2.8	0.88	0.5	3.0	9403.7–7498.3; 6102–4597.7
15:0*ai*	1D+SNV	3	0.81	1.3	2.3	0.80	1.0	2.4	6102–5446.3
17:0*ai*/13*t*-16:1	1D+SNV	3	0.84	3.6	2.5	0.85	3.0	2.6	9403.7–8447.2; 5778–5446.3; 4605.4–4420.3
**Σ MUFA**	1D	4	0.95	177.0	4.4	0.95	169.0	4.3	7506–5446.3; 4605.4–4242.9
**Σ 16:1-** ***cis***	1D	5	0.91	18.2	3.4	0.90	15.0	3.3	6102–4597.7
**Σ 18:1-** ***cis***	1D	4	0.95	141.0	4.6	0.95	130.0	4.6	6102–4597.7
9*c*-14:1	1D	5	0.85	4.1	2.6	0.85	4.2	2.6	7506–4242.9
7*c*-16:1	1D+SNV	3	0.83	1.2	2.4	0.84	0.9	2.6	7506–6094.3; 4605.4–4242.9
9*c*-16:1	1D	4	0.86	17.4	2.7	0.83	19.7	0.3	6102–5770.3; 4428–4242.9
11*c*-16:1	SNV	6	0.84	0.5	2.5	0.80	0.5	2.3	5454–4242.9
13*c*-16:1	1D	3	0.78	0.3	2.1	0.75	0.3	2.0	9403.7–7498.3; 6102–4242.9
6*c*-18:1/8*c*-18:1	1D	3	0.78	0.1	2.1	0.75	0.1	2.1	7506–6796.3; 5454–4242.9
9*c*-17:1	1D+SNV	2	0.84	2.8	2.5	0.91	2.0	3.4	6102–5446.3
9*c*-18:1/10*c*-18:1	1D	3	0.95	145.0	4.3	0.96	107.0	4.8	6102–5168.6
11*c*-18:1	1D	5	0.90	4.7	3.2	0.92	3.6	3.5	6102–5446.3; 4605.4–4242.9
12*c*-18:1	SNV	2	0.72	0.6	1.9	0.71	0.5	1.9	7506–4242.9
13*c*-18:1	1D	5	0.88	1.6	2.9	0.86	1.1	2.7	7428.9–5446.3; 4428–4242.9
15*c*-18:1	SNV	5	0.85	0.7	2.6	0.84	0.6	2.6	9403.7–7498.3; 6102–4597.7
10*c*-20:1	NSDP	6	0.90	0.4	3.1	0.90	0.4	3.2	3594.9–1289.5
11*c*-20:1	1D	2	0.88	0.6	2.9	0.87	0.7	2.8	6102–5770.3
**Σ 16:1-** ***trans***	1D	2	0.82	1.0	2.4	0.81	1.0	2.3	9403.7–7498.3; 5778–5446.3
**Σ 18:1-** ***trans***	1D	3	0.88	14.4	2.9	0.85	11.7	2.6	9403.7–7498.3; 4852.3–4242.9
6*t*-16:1/8*t*-16:1	SNV	3	0.71	0.1	1.9	0.71	0.1	1.9	9403.7–6094.3; 5454–4242.9
9*t*-16:1	SNV	1	0.05	0.9	1.0	0.87	0.7	2.8	6102–2446.3
10*t*-16:1	SNV	2	0.35	0.1	1.2	0.39	0.1	1.3	9403.7–8447.2; 5778–5446.3; 4428–4242.9
11*t*-16:1/12*t*-16:1	1D	3	0.81	0.4	2.3	0.83	0.4	2.4	6102–4597.7
14*t*-16:1/12c-16:1	SNV	2	0.62	0.2	1.6	0.61	0.2	1.7	9403.7–7498.3; 4605.4–4242.9
6*t*-18:1/8*t*-18:1	1D	4	0.83	0.8	2.4	0.87	0.7	2.8	9403.7–7498.3; 6102–4242.9
9*t*-18:1	1D	3	0.90	0.9	3.2	0.90	0.7	3.2	9403.7–4242.9
10*t*-18:1	1D	3	0.92	1.0	2.3	0.82	0.9	2.4	9403.7–6094.3; 5454–4242.9
11*t*-18:1	1D	7	0.74	12.7	2.0	0.74	10.7	2.1	7506–6094.3
12*t*-18:1	NSDP	7	0.83	1.2	2.4	0.84	1.3	2.5	3594.9–12,489.5
13*t*-18:1/14*t*-18:1	1D+SNV	5	0.81	2.0	2.3	0.82	1.8	2.4	5778–5446.3; 4605.4–4420.3
15*t*-18:1	1D	3	0.78	0.6	2.1	0.78	0.4	2.1	5454–4242.9
16*t*-18:1/*c*,*t*-18:2	SNV	8	0.90	1.1	3.2	0.89	0.9	3.0	7506–5446.3; 4605.4–4242.9
**Σ PUFA**	SNV	5	0.69	17.9	1.8	0.65	1.7	1.1	9403.7–7498.3; 5454–4242.9
**Σ n-6**	NSDP	4	0.43	15.1	1.3	0.44	10.3	1.3	3594.9–12,489.5
18:2n-6	NSDP	5	0.49	10.2	1.4	0.52	7.4	1.5	3594.9–12,489.5
18:3n-6	COE	4	0.46	0.1	1.4	0.44	0.2	1.3	3594.9–12,489.5
20:2n-6	NSDP	5	0.34	0.2	1.2	0.30	0.2	1.2	3594.9–12,489.5
20:3n-6	NSDP	2	0.16	1.2	1.1	0.18	0.9	1.1	3494.3–12,498.5
20:4n-6	1D	2	0.42	3.9	1.3	0.44	2.5	1.4	9403.7–7498.3; 4605.4–4242.9
22:4n-6	NSDP	5	0.45	0.4	1.4	0.47	0.3	1.4	3594.9–12,489.5
**Σ n-3**	NSDP	5	0.47	11.0	1.4	0.50	7.8	1.4	3594.3–12,489.5
18:3n-3	NSDP	4	0.22	7.6	1.1	0.41	5.2	1.3	3594.9–12,489.5
20:3n-3	1D+SNV	8	0.69	0.2	1.8	0.66	0.2	1.7	9403.7–6094.3; 4605.4–4242.9
20:4n-3	NSDP	8	0.31	0.9	1.2	0.36	0.7	1.2	3594.9–12,489.5
20:5n-3 (EPA)	NSDP	8	0.63	2.3	1.6	0.64	1.9	1.7	3594.9–12,489.5
22:5n-3 (DPA)	1D+SNV	5	0.40	1.6	1.3	0.38	1.4	1.3	8454.9–7498.3;6102–5446.3; 4605.4–4242.9
22:6n-3 (DHA)	NSDP	4	0.33	0.5	1.2	0.33	0.5	1.2	3594.9–12,489.5
**Σ DMA**	NSDP	5	0.71	0.2	1.9	0.70	0.2	1.8	3594.9–12,489.5
**Σ CLA**	1D	2	0.83	5.1	2.4	0.84	4.9	2.5	6102–5770.3
**Σ CLA-** ***trans,trans***	NSDP	5	0.74	0.5	2.0	0.76	0.4	2.0	3594.9–12,489.5
**Σ CLA-** ***cis,trans***	NSDP	5	0.81	5.0	2.3	0.80	4.2	2.3	3594.9–12,489.5
7*t*,9*c*-18:2	NSDP	6	0.77	0.2	2.1	0.75	0.2	2.1	3594.9–12,489.5
9*c*,11*t*-18:2	NSDP	6	0.78	3.9	2.1	0.79	2.9	2.3	3594.9–12,489.5
9*t*,11*c*-18:2	NSDP	1	0.55	0.1	1.5	0.57	0.1	1.5	3594.9–12,489.5
11*c*,13*t*-18:2/21:0	NSDP	5	0.65	0.1	1.7	0.64	0.1	1.8	3594.9–12,489.5
11*t*,13*t*-18:2	NSDP	5	0.74	0.3	2.0	0.76	0.2	2.1	3594.9–12,489.5
12*t*,14*t*-18:2	NSDP	5	0.67	0.1	1.8	0.69	0.1	1.8	3594.9–12,489.5
7*t*,9*t/*10*t*,12*t*-18:2	NSDP	5	0.74	0.2	2.0	0.73	0.1	1.9	3594.9–12,489.5
**Σ NC-dienes**									
9*c*,13*t*-18:2/8*t*,12*c*-18:2	SNV	4	0.87	1.2	2.8	0.87	0.1	2.9	6102–5770.3; 4605.4–4242.9
8*t*,13*c*-18:2	NSDP	5	0.83	0.7	2.4	0.84	0.6	2.5	3594.9–12,489.5
9*t*,12*c*-18:2	NSDP	5	0.73	0.2	1.9	0.72	0.2	1.9	3594.9–12,489.5
11*t*,15*c-18:2*/10*t*,15*c*-18:2	SNV	1	0.71	1.5	1.9	0.72	1.5	1.9	6102–5445.3; 4605.4–4242.9
9*c*,15*c*-18:2	NSDP	6	0.87	0.6	2.7	0.86	0.6	2.7	3594.9–12,489.5
**Σ NC-trienes**									
9*c*,11*t*,15*c*-18:3/20:3n-9	NSDP	5	0.54	0.7	1.5	0.55	0.7	1.5	3594.9–12,489.5
9*c*,11*t*,15*t*-18:3	1D	1	0.77	0.2	2.1	0.77	0.2	2.1	6102–5770.3; 4428–4242.9

Σ SFA: saturated fatty acids; Σ BCFA: branched-chain saturated fatty acid; Σ DMA: dimethyl acetal; Σ MUFA: Monounsaturated fatty acids; Σ PUFA: polyunsaturated fatty acids; ΣCLA: Conjugated linoleic acid; Σ NC: non-conjugated;; NSDP: No spectral data preprocessing; COE: Constant offset elimination; 1D: first-order derivative; 1D+SNV: standard normal variate combined with first-order derivative; RMSEE: Root mean square error of estimation; RMSEP: Root mean square error of prediction; RPD: residual predictive deviation.

**Table 3:** Statistical comparison between GC and NIRS values for fatty acids predicted by the NIRS model, using paired *t*-test and agreement metrics.

Chemical Component	*p*-Value	Residual Mean	RMSE	R^2^	Bias	Slope
**Σ FAME**	0.62	136.61	273.00	0.01	−137.00	0.98
**Σ SFA**	0.79	−37.64	152.00	0.00	37.60	0.86
10:0	0.65	0.08	0.40	0.01	−0.08	0.45
12:0	0.27	0.24	0.52	0.04	−0.24	0.56
14:0	0.49	−7.06	20.70	0.02	7.06	0.58
15:0	0.00 *	−11.92	730.00	0.45	701.00	0.01
16:0	0.50	51.68	763.00	0.02	−740.00	14.57
17:0	0.00 *	15.40	16.30	0.65	−15.40	1.15
18:0	0.42	41.87	80.70	0.02	−41.90	0.76
19:0	0.72	−0.13	0.94	0.01	0.13	0.30
20:0	0.29	0.31	0.56	0.04	−0.31	0.71
22:0	0.34	−0.10	0.32	0.03	0.10	0.20
15:0*iso*	0.02 *	0.17	1.92	0.00	−0.17	0.43
17:0*iso*	0.69	0.46	2.71	0.01	−0.46	0.48
18:0*iso*	0.48	0.24	0.79	0.02	−0.24	0.52
15:0*ai*	0.58	0.48	2.40	0.01	−0.48	0.30
17:0*ai*/13*t*-16:1	0.08	3.64	5.96	0.11	−3.64	0.48
**Σ 16:1-** ***cis***	0.63	−5.40	19.80	0.01	5.40	0.75
**Σ 18:1-** ***cis***	0.81	26.40	83.40	0.00	−26.40	1.03
9*c*-14:1	0.36	−2.62	7.17	0.03	2.62	0.44
7*c*-16:1	0.55	0.32	1.29	0.01	−0.32	0.61
9*c*-16:1	0.91	1.21	19.50	0.00	−1.21	0.60
11*c*-16:1	0.97	0.02	0.93	0.00	−0.02	0.41
9*c*-17:1	0.38	1.30	3.07	0.03	−1.30	0.68
9*c*-18:1/10*c*-18:1	0.98	2.14	75.30	0.00	−2.14	1.05
11*c*-18:1	0.65	−1.11	2.75	0.01	1.11	0.90
12*c*-18:1	0.55	−0.24	1.29	0.01	0.24	0.16
13*c*:18:1	0.21	−1.13	1.66	0.06	1.12	0.70
15*c*-18:1	0.76	−0.19	1.99	0.00	0.19	0.17
11*c*-20:1	0.99	−0.01	0.91	0.00	0.01	0.55
**Σ 16:1-** ***trans***	0.00 *	1.55	2.21	0.33	−1.55	−0.02
**Σ 18:1-** ***trans***	0.40	8.91	24.50	0.03	−1.55	−0.02
9*t*-16:1	0.00 *	1.02	1.25	0.55	−1.02	−0.15
11*t*-16:1/12*t*-16:1	0.04 *	0.32	0.62	0.14	−0.32	0.20
9*t*-18:1	0.68	0.25	0.92	0.01	−0.25	0.72
10*t*-18:1	0.08	−1.63	3.11	0.10	1.63	0.23
11*t*-18:1	0.12	8.34	16.80	0.09	−8.33	0.34
12*t*-18:1	0.37	−0.75	1.73	0.03	0.75	0.50
13*t*-18:1/14*t*-18:1	0.53	1.00	3.20	0.01	−1.00	0.60
16*t*-18:1/*cis,trans*-18:2	0.71	0.31	1.87	0.01	−0.31	0.60
**Σ PUFA**	0.08	13.43	30.20	0.10	−13.40	0.03
**Σ n-6**	0.97	−0.27	23.90	0.00	0.27	−0.01
18:2n-6	0.51	−3.22	17.00	0.02	3.22	0.07
18:3n-6	0.01 *	−0.25	0.38	0.24	0.25	0.12
20:2n-6	0.15	−0.29	0.74	0.07	0.29	0.05
20:3n-6	0.39	−0.37	1.56	0.03	0.37	0.05
20:4n-6	0.45	−1.11	4.72	0.02	1.11	0.16
22:4n-6	0.03 *	−0.58	1.04	0.15	0.58	0.10
**Σ n-3**	0.00 *	21.52	24.70	0.66	−21.50	−0.32
18:3n-3	0.00 *	14.20	14.90	0.81	−14.20	0.08
20:3n-3	0.16	0.13	0.35	0.07	−0.13	0.05
20:4n-3	0.00 *	1.15	1.34	0.59	−1.15	−0.03
20:5n-3 (EPA)	0.16	1.22	3.09	0.07	−1.22	0.18
22:5n-3 (DPA)	0.74	−0.20	2.37	0.00	0.20	−0.11
22:6n-3 (DHA)	0.05 *	−0.49	1.00	0.13	0.49	0.00
**Σ CLA**	0.00 *	9.33	10.40	0.47	−9.33	1.47
**Σ CLA-** ***cis,trans***	0.00 *	8.69	9.59	0.48	−8.69	1.55
9*c*,13*t*-18:2/8*t*,12*c*-18:2	0.92	0.06	0.91	0.00	−0.06	0.83
9*c*,15*c*-18:2	0.39	0.28	0.61	0.03	−0.28	0.78
8*t*,13*c*-18:2	0.87	−0.05	0.52	0.00	0.05	0.91
9*t*,12*c*-18:2	0.57	0.05	0.28	0.01	−0.05	0.15
11*t*,15*c*-18:2/10*t*,15*c*-18:2	0.34	0.82	2.48	0.03	−0.82	0.30

*p*-values from paired *t*-tests, residual means (GC-NIRS), RMSEP (Root Mean Square Error of Prediction), R^2^p (coefficient of determination for prediction), bias (systematic error), and slope of the regression line (GC vs. NIRS). (*) significant differences (*p* < 0.05) between GC and NIRS values. Σ FAME: Fatty acids methyl ester; Σ SFA: saturated fatty acids; Σ PUFA: polyunsaturated fatty acids; Σ CLA: Conjugated linoleic acid; Σ NC: non-conjugated.

## Data Availability

The original contributions presented in this study are included in the article. Further inquiries can be directed to the corresponding author.
